# Inflamed *In Vitro* Retina: Cytotoxic Neuroinflammation and Galectin-3 Expression

**DOI:** 10.1371/journal.pone.0161723

**Published:** 2016-09-09

**Authors:** Patrik Maximilian Bauer, Marina Castro Zalis, Hodan Abdshill, Tomas Deierborg, Fredrik Johansson, Ulrica Englund-Johansson

**Affiliations:** 1 Dept. of Biology, Sec. Functional Zoology, Lund University, Lund, Sweden; 2 Dept. Clinical Sciences in Lund, Div. Ophthalmology, Lund University, Lund, Sweden; 3 Dept. Experimental Medical Science, Experimental Neuroinflammation Laboratory, Lund University, Lund, Sweden; Universidade de Sao Paulo, BRAZIL

## Abstract

**Background:**

Disease progression in retinal neurodegeneration is strongly correlated to immune cell activation, which may have either a neuroprotective or neurotoxic effect. Increased knowledge about the immune response profile and retinal neurodegeneration may lead to candidate targets for treatments. Therefore, we have used the explanted retina as a model to explore the immune response and expression of the immune modulator galectin-3 (Gal-3), induced by the cultivation *per se* and after additional immune stimulation with lipopolysaccharide (LPS), and how this correlates with retinal neurotoxicity.

**Methods:**

Post-natal mouse retinas were cultured in a defined medium. One group was stimulated with LPS (100 ng/ml, 24 h). Retinal architecture, apoptotic cell death, and micro- and macroglial activity were studied at the time of cultivation (0 days *in vitro* (DIV)) and at 3, 4 and 7 DIV using morphological staining, biochemical- and immunohistochemical techniques.

**Results:**

Our results show that sustained activation of macro- and microglia, characterized by no detectable cytokine release and limited expression of Gal-3, is not further inducing apoptosis additional to the axotomy-induced apoptosis in innermost nuclear layer. An elevated immune response was detected after LPS stimulation, as demonstrated primarily by release of immune mediators (*i*.*e*. interleukin 2 (IL-2), IL-6, KC/GRO (also known as CLCX1) and tumour necrosis factor-α (TNF-α)), increased numbers of microglia displaying morphologies of late activation stages as well as Gal-3 expression. This was accompanied with increased apoptosis in the two additional nuclear layers, and damage to retinal gross architecture.

**Conclusion:**

We demonstrate that an immune response characterized by sustained and increased release of cytokines, along with an increase in Gal-3 expression, is accompanied by significant increased neurotoxicity in the explanted retina. Further investigations using the current setting may lead to increased understanding on the mechanisms involved in neuronal loss in retinal neurodegenerations.

## Introduction

The retina constitutes a remote extension of the brain, located at the back of the eye, and is a light-sensitive neural structure enabling vision. Retinal neurodegenerative diseases, such as glaucoma, diabetic retinopathy and age-related macular degeneration (AMD), lead to loss of neurons, resulting in progressive loss of visual function.

Neuroinflammation is an important regulator of the disease progression in several brain neurodegenerative diseases [1–3]. The immune response of the retina is similar to other brain regions, involving activation of microglial cells, macroglial cells, *i*.*e*. astrocytes and Müller cells, vascular pericytes as well as infiltration of blood-borne macrophages [4–6]. Microglial cells are the resident macrophages of the retina; when activated by disease, injury or pathogens, microglia release a large array of immune mediators [7–9], proliferate, migrate toward damaged sites and transform into amoeboid phagocytic cells. Uncontrolled microglia activation is widely reported to contribute to disease progression in Alzheimer’s disease, Parkinson’s disease (PD) and multiple sclerosis [1–3]. In parallel, increasing reports suggest that microglial cells are a crucial component in the acute and chronic disease phases of retinal degeneration [10–16]. For example, changed communication between retinal neurons and microglia, astrocytes or oligodendrocyte is seen in many brain conditions and was recently described as an early pathological sign in experimental glaucoma studies [5, 17].

Lately, we, along with others, have demonstrated the role of one meditator of microglia activation, galactin-3 (Gal-3), in a range of neurodegenerations, including PD, brain ischemia, diabetic retinopathy and light-induced retinal degeneration [18–21]. Gal-3 is a member of the β-galactoside-binding lectin family defined by their typical carbohydrate recognition domains (CRDs) [22, 23]. Gal-3 plays a role in different biological activities, including cell adhesion, proliferation, clearance, apoptosis, cell activation, cell migration, phagocytosis and inflammatory regulation [24–29]. Recently, Gal-3 upregulation in glial cells was associated with the axonal degenerative process in a glaucoma model *in vivo*, and was described to have a detrimental role in hypoperfusion-induced retinal degeneration [30, 31]. However, retinal neuroinflammation is a rather new research field, and its relevance for disease progression is still largely unexplored.

A model extensively used in the investigation of mechanisms of retinal disease pathology is the organotypic cultured retina [32, 33]. Retinal cultures from different ages, species as well as from disease models have been characterized and used in studies ranging from basic developmental studies to testing response to different therapies [34–37]. The cultivation process *per se* leads to a progressive degeneration of the retinal ganglion cells (RGC) caused by axotomy of the optic nerve [38, 39]; but also loss of neurons in the more outer layers of the retina [32, 40]. Using immunohistochemistry, Engelsberg *et al* described the microglial activation and apoptotic neurodegeneration after explantation of the post-natal rat retina, beginning in the inner retina and advancing through the other retinal layers over time [40]. Additionally, transient microglia activation and sustained macroglia activation, including a transient release of low levels of common cytokine, *i*.*e*. tumour necrosis factor-α (TNF-α) and interleukin- 6 (IL-6), were reported using a similar culture protocol [33].

Manipulation of microglial activation in retinal culture has been described by several laboratories [33, 41] through stimulation with lipopolysaccaride (LPS) [42, 43] or pharmacological inhibition of microglial activation with *e*.*g*. minocycline [44]. LPS, is a bacterial component, commonly used to elevate microglial activation with a subsequent often reported neurotoxic effect, presumably mediated by microglia [45].

Our laboratory has established and extensively used a protocol for the diseased and normal post-natal mouse retina, where a specified serum-free media allows the culture of well-preserved retinas up to 4 weeks [46, 47]. This culture protocol gives the opportunity to assess treatment effects without the interference of unknown serum factors. We believe that this model can be utilized to further elucidate hallmarks and mechanisms of retinal neuroinflammation, and its relation to retinal function and health. Therefore, we wanted to explore the immune response profile including Gal-3 expression (known to contribute to the full activation of the LPS receptor toll-like receptor 4 (TLR4) [19]) induced by the cultivation process *per se* and after additional stimulation with LPS and its correlation with retinal neurotoxicity using our retina culture protocol.

## Material and Methods

### Animals

Animals were kept under conditions with standard white 12 h cycling lightning, free access to food and water, and used irrespectively of gender. In-house bred C3H/HeA wild-type (wt) mice [48] were used in the study. Mouse retinal tissue was taken at post-natal day 7 (PN7). Animal handling was performed in accordance with approved guidelines of the Ethical Committee of Lund University, the Institute for Laboratory Animal Research (Guide for the Care and Use of Laboratory Animals, Malmö-Lund Ethical Committee in Sweden), and the ARVO regulations for the use of animals in ophthalmic and vision research.

### Retinal explant culture

Animals were sacrificed by an overdose of CO₂. Eyes were enucleated; and the anterior segment, the vitreous body and the sclera removed. The neural retina with pigmented epithelium was explanted onto a Millicell-PCF 0.4 μm culture plate inserts (Millipore, Merck, Solna, Sweden) with the vitreal side oriented upwards. Retinal explants where cultured in R16 culture medium (Invitrogen Life Technologies, Paisley, UK; 07490743A). Explants were allowed to adjust to culture conditions for two days, before receiving fresh R16 medium and to selected groups addition of LPS (100 ng/ml) for 24 h. Conditioned media was collected from LPS- exposed retinas and corresponding controls at 2, 3, 4 and 7 DIV days.

### Tissue processing

For histological staining the retinas were fixed for 2 h in 4% paraformaldehyde and then embedded in a Yazulla medium (30% egg albumin and 3% gelatin in distilled water). Cryosections of 12–16 μm were cut, mounted onto chrome-alum coated glass slides, and stored at -20°C.

### Hematoxylin-eosin staining and gross morphological analysis

For gross morphological analysis, specimens was stained with Hematoxylin-eosin (Htx-Eosin), dehydrated, and cover slipped using Pertex mounting media (HistoLab, Sweden). Gross as well as detailed morphological analysis was performed using light microscopy (Nikon, Tokyo, Japan). Eight-ten sections per specimen representing the entire retina were included (n = 4–6 retinas/group). Evaluation of gross morphology was made with a ranking system divided into five different categories:

- Layering (0 = normal layering, 0.5 = minor deformation, 1 = major deformation)
- Fold formation (0 = no folds, 0.5 = few folds, 1 = many folds)
- Rosette formation (0 = no rosettes, 0.5 few rosettes, 1 = many rosettes)
- Nuclear layer tissue architecture (0 = normal, 0.5 = small and few disseminated regions, 1 = large and many disseminated regions)
- Pyknotic nuclei (0 = <10, 0.5 = 10–50, 1 = >50)

### TUNEL assay and quantification of TUNEL-positive cells

Apoptotic cells were labeled using the in situ dUTP nick end labeling assay (TUNEL; Roche, Mannheim, Germany). Retinal sections were stained according to manufacturer’s instructions, and cover slipped using 4′6-diamidino-2-phenylindole(DAPI)-containing Vectashield mounting medium (Vector Laboratories, Burlingame, CA, USA).

Quantification of TUNEL-positive cells, DAPI-positive cells and area measurement was performed using ImageJ (National Institutes of Health, Washington, D.C.). After LPS exposure and in corresponding controls TUNEL-positive cells were counted in all neuronal nuclear layers, *i*.*e*. the GCL, INL and ONL, respectively. Eight sections per specimen from four separate slides representing the entire retina were included (n = 3–6 retinas/group).

### Fluorescent immunostaining- Iba1, ED1, Gal-3, Ki67 and GFAP and quantifications

Sections were rinsed and pre-incubated in PBS-T (PBS, 0.25% Triton X-100, 1% bovine serum albumin (BSA)) for 1 h at room temperature (RT). Sections were then incubated with primary antibodies, rabbit anti-glial fibrillary acidic protein (GFAP, 1:2000, DAKO Cytomation, Glostrup, Denmark), rabbit anti-Iba1 (1:200, WAKO, Tokyo, Japan), rat anti-mouse ED1 (CD68, 1:1000, Nordic Biosite, Täby, Sweden), rat anti-mouse Gal-3 (1:500 WAKO, Tokyo, Japan), mouse anti-NeuN (1:100, Chemicon International, Temecula, CA, USA) or anti-goat Ki67 (1:100, Millipore, Temecula, CA, USA) at 4°C overnight, before subsequent rinsing and incubation in secondary antibodies for 2 h. Secondary antibodies used were Texas Red-conjugated donkey anti-rabbit antibody (1:200; Abcam, Cambridge, UK), Alexa Fluor 488 goat anti-rabbit IgG (Molecular Probes, Inc. Eugene, OR, USA) and Alexa Fluor 564 goat anti-rat (Molecular Probes, Inc. Eugene, OR, USA). Both primary and secondary antibodies were diluted in PBS-T. For counterstaining of nuclei, the sections were cover-slipped using Vectashield mounting media containing DAPI (Vector Laboratories, Inc. Burlingame. CA, USA).

Macroglial response was analyzed by qualitative gross- as well as detailed analysis, in eight sections per specimen (n = 4–6 retinas/group) from four separate slides representing the entire retina for macroglial cell activation (astrocytes and Müller cells) using GFAP staining and fluorescence microscopy (Nikon Eclipse E800, Tokyo, Japan).

Total numbers of microglial cells were quantified, using the marker Iba1 in six sections per specimen from 3 separate slides representing the entire retina. The level of microglia activation was assessed by morphological changes as well as expression of two different markers of activation, *i*.*e*. ED1 and Gal-3. The following morphological classification for activation stage was used;

*Ramified* (round cell body, long branched processes) *=* resting stage

*Round* (round cell body, no processes) = active stage

*Amoeboid* (irregular/ellipsoid cell body, no process) only found in GCL layer = active stage

The numbers of ED1- and Gal-3-positive cells, respectively, of total number of Iba1 was quantified, 6–8 sections per specimen (n = 3–6 retinas/group) from 3–4 separate slides representing the entire retina. The ED1- and Gal-3-positive cells, respectively, were also morphologically classified as described above.

### ELISA

Conditioned media was collected at 2, 3, 4 and 7 DIV (n = 3 samples/group). A Electrochemoluminiscence ELISA was performed using the V-PLEX Plus Proinflammatory Panel 1 (mouse) kit (Mesoscale discoveries, Rockville, Maryland USA). The following immune mediators were analysed, Interferon gamma, Interleukin-1 beta, Interleukin-2, Interleukin-4, Interleukin-5, Interleukin-6, Interleukin-10, Interleukin-12p70, KC/GRO and tumor necrosis factor alpha. A pre-coated plate was used with capture antibodies on independent and well-defined spots in a 10-spot MULTI-SPOT^®^ plate. All CV-values (coefficient of variation) above 35 were closer investigated and the values beneath detection level or/and out of calibration range were removed.

### Statistical analysis

Statistical analysis was performed using SPSS 22 (IBM, NY, USA) software. For ANOVA analysis, 2- and 3 way ANOVA tests were used. A chi-test was used to analyze the ranking results in the gross morphological analysis. Correlation analysis was also performed and all results are presented as mean±standard deviation (StDev), and p<0.05 were considered significant.

## Results and Discussion

### Experimental design

We use post-natal mouse retina from day 7 in our study, as at this time-point all retinal layers are developed [49]. As we wanted to do a detailed histological study, we utilized an organotypic culture model extensively used in our laboratory, which provides well-preserved laminar retinal architecture over a long period of time in culture. The model comprises whole mount retinal culture in defined serum-free media [50]. Importantly, post-natal rodent retinas possess a microglial population that has spread throughout the entire retina (from the central part to the ora serrata) resembling that of an adult and formed quiescent, ramified morphology at about the age used here [32, 33].

We explored how the cultivation process as well as LPS administration influences retinal cells viability and macro- and microglia behavior. Only retinas that had attached well to the culture membrane within 48 h were included in the analysis. At 2 DIV, selected retina cultures were exposed to LPS (100 ng/ml) for 24 h, as this LPS protocol has previously been reported to elevate microglia activation [33].

### LPS treatment caused retinal gross morphological changes

Distortion of the well-lineated retinal structure as well as presence of pyknotic cells are clear signs of decreased retinal viability. Pyknotic nuclei, having condensed chromatin, are often found in cells undergoing necrosis or apoptosis [51]. In our previous studies we have successfully used a ranking protocol to assess gross morphological changes in the retinas as a tool to evaluate cytotoxic effects; *i*.*e*. after nanoparticle exposure to the cultured mouse retina [52] and post-seizures rat [53].

**Fig 1:**
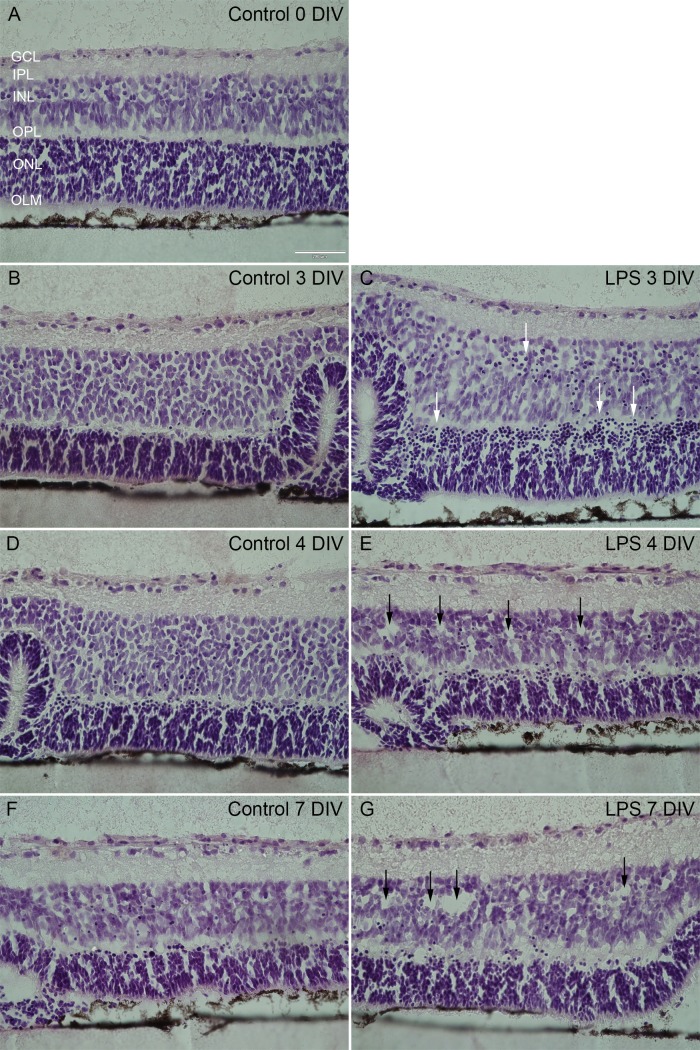
Retinal gross morphology shown by Htx-eosin staining. At the time of explantation, *i*.*e*. 0 DIV, normal retinal lamination, including three distinct nuclear layers and two synaptic layers with no folds, no rosettes and absence of pyknotic cells were found (A). Cultivation *per se* did not induce any architectural changes until at 7 DIV (cf. A, B, D with F). LPS exposed retinas displayed gross morphological changes at all time-points, with signs of cytotoxicity, *i*.*e*. pyknotic nuclei mainly in the INL and (see arrows in C) disseminated tissue especially in the INL (see arrows in E and G). GCL = ganglion cell layer, IPL = inner plexiform layer, INL = inner nuclear layer, OPL = outer plexiform layer, ONL = outer nuclear layer, OLM = outer limiting membrane. Scale bar: 200 μm.

First we evaluated the retinal response to the culture process at a gross morphological level up to 7 DIV, using Htx-eosin histological staining. Retinas immediately fixed after seeding at the culture membranes served as a baseline control, and displayed normal *in vivo*-resembling lamination with three distinct nuclear layers and two synaptic layers with no folds, no rosettes, no tissue changes within the nuclear layers or presence of cells with pyknotic nuclei (Fig 1A). No significant alterations in gross morphology were found up to 7 DIV. However, occasional pyknotic cells were seen from 1 DIV up to 7 DIV (Fig 1A, 1B, 1D and 1F).

We then explored whether LPS administration had any effect on the retinal gross morphology. Immediately after the removal of LPS (acute response) we observed a larger fraction of pyknotic nuclei, especially in the inner nuclear layer (INL) and outer plexiform layer (OPL). When compared to control for these layers, we found a strong difference (19% vs 80% affected, n = 4-6/group, p = 0.067) (Fig 1C). In addition to pyknotic nuclei, at 4 and 7 DIV, disseminated nuclear layers, characterized by frequent cell-free areas, mainly in INL were identified in the LPS group (Fig 1E and 1G). However, only at 2 days post LPS-treatment was a significant difference observed in nuclear layer structure, with 60% of LPS-treated retinas affected compared to no observable difference in control retinas (n = 5/group, p = 0.038). At 5 DIV we observed a non-significant effect on gross morphology between control and corresponding LPS-group. This may be explained by the trend toward changes in gross morphology in the control group over time in culture. Both the control- and treatment group showed the same, relatively low amount of rosette and folding formations at all time-points, indicating these as common phenomena while culturing retinas *in vitro*.

Notably, tissue damage at the gross morphological level is often related to the chronic phase of neuroinflammation [54, 55]. The rather acute tissue distortion seen here may be explained by the retina being more vulnerable when removed from its natural environment.

### LPS increased numbers of apoptotic cells

**Fig 2:**
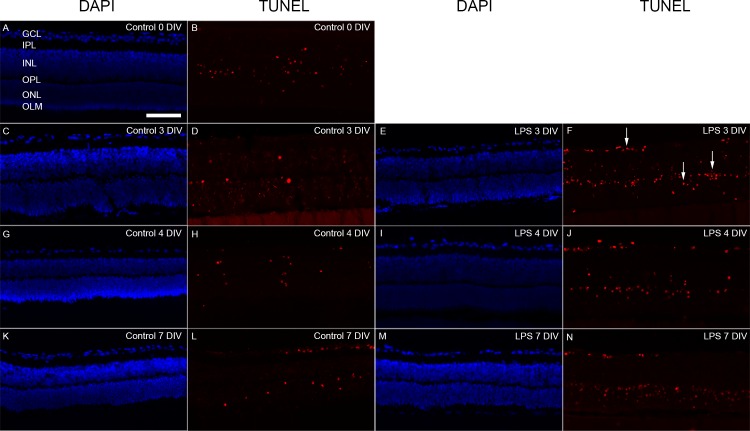
Apoptotic cell death detected with TUNEL staining. At 0 DIV, apoptotic cells (TUNEL-positive (red)) were primarily found in the INL (A, B). Nuclei were labelled with DAPI (blue). Over time in culture, moderate number of TUNEL-positive cells was seen in the non-treated group, in all nuclear layers, the GCL, INL and ONL (D, H, and L). In the corresponding age-matched LPS exposed groups, larger numbers of TUNEL-positive cells were found, especially at 3 DIV (F, arrows). Abundant numbers of TUNEL-labelled cells were also found at 4 and 7 DIV (J and N, respectively). GCL = ganglion cell layer, IPL = inner plexiform layer, INL = inner nuclear layer, OPL = outer plexiform layer, ONL = outer nuclear layer, OLM = outer limiting membrane. Scale bar: 200 μm.

We next investigated apoptotic cell death dynamics using TUNEL labeling. In control retinas, TUNEL-positive cells were found at all time-points and in all nuclear layers, but to different degrees (Fig 2).

**Fig 3:**
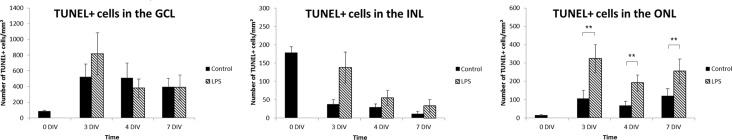
Quantification of apoptotic cells. The graphs show numbers of TUNEL-positive cells in the GCL, INL and ONL, in controls- and LPS-treated groups. ANOVA analysis was performed for comparison between the LPS treated group and the control groups. Data are given as mean±StDev (n = 4-6/group), **p<0.01.

At 0 DIV, resembling *in vivo* PN7 retinas, abundant numbers of apoptotic cells were found in the INL, but only occasionally detected in the GCL and ONL (Fig 3). This can be explained by the apoptosis-dependent remodeling reported to occur at PN7 [49], especially in the INL. Apoptosis has been reported to cease *in vivo* between PN10-14 [49], we observe a similar trend in cultured retinas, with only a limited number of apoptotic cells identified over time in the INL (Fig 3). Moreover, in line with Engelsberg *et al* (2004), we also found a slight increase in TUNEL-labeled cells in the ONL at 7 DIV [40].

In the GCL, and in line with previous reports [40], an acute and massive loss of RGCs caused by axotomy, was found in the control retinas. Quantification using the marker NeuN for neurons in the GCL, *i*.*e*. RGC and amacrine cells, revealed a loss of 50% at 2 DIV (data not shown). Thereafter, quantification shows an equal number of TUNEL-labeled cells in the GCL at all time-points studied (Fig 3). In addition, our results are in parallel with previous studies demonstrating no further loss of the remaining GCL neurons up to 14 DIV, the latest time-point analyzed here (data not shown and [56]).

LPS-treated retinas at 3 DIV showed an increase in TUNEL-labeled cells in all nuclear layers (Fig 3). Although nearly twice the numbers of apoptotic cells were found in the GCL and INL, a significant increase was only observed in the ONL (Fig 3). In the GCL and INL this increase was transient and disappeared at later time-points. In contrast, in the ONL the number of TUNEL-positive cells remained significantly higher than in the control at later time-points (Fig 3).

In selected specimens from all groups we studied whether the microglia cells die from LPS treatment by using double-staining with ED1 and TUNEL. However, no apoptotic ED1-expressing microglial cells could be found (data not shown). Hence, we believe that most likely the TUNEL-positive cells in the nuclear layer are of a neuronal origin.

In summary, our cultivation protocol induces massive cell death in the GCL, caused mainly by axotomy and a low rate of apoptosis in the INL and ONL. Secondly, LPS treatment triggered increased apoptotic cell loss, transient in the GCL and INL, but sustained in the ONL. This might indicate that photoreceptors are more vulnerable to inflammation caused by LPS [32, 33, 57].

### LPS elevated macroglial cell activation

Macroglial cells of the retina are found within the nerve fiber layer (NFL) (astrocytes), while Müller cell bodies are localized in the INL with their fibers spanning the entire retina, from inner limiting membrane (ILM) to the outer limiting membrane (OLM). In normal conditions in the mammalian retina, GFAP is only expressed by the astrocytes in the inner retina. Up-regulation of GFAP in astrocytes and especially in Müller cells is a well-described hallmark of a damaged/stressed retina [58], [59].

**Fig 4:**
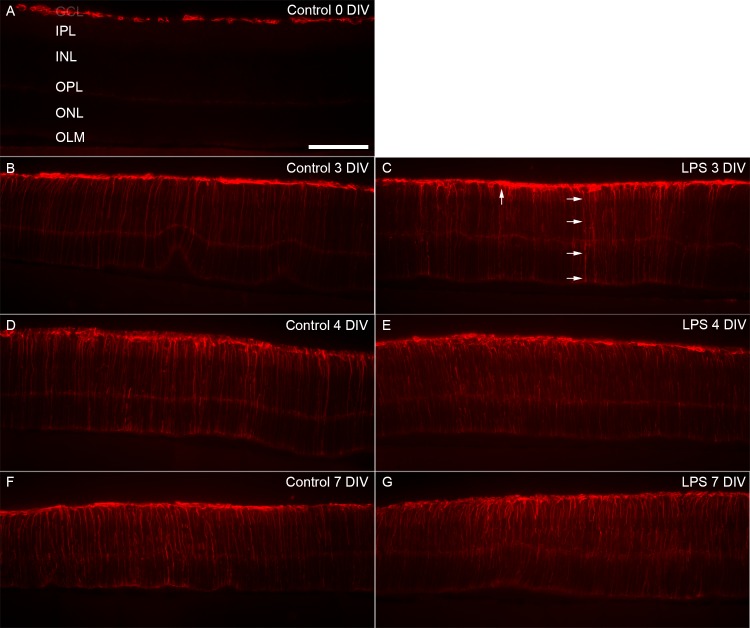
Macroglial activity detected by GFAP- staining. GFAP-immunohistochemistry was used to label the macroglial cells of the retina, *i*.*e*. astrocytes and Müller cells. At 0 DIV, GFAP-staining was primarily detected in the NFL (A). An increase in GFAP-staining was detected at 3, 4, and 7 DIV (B, D and F); with Müller cell up-regulation of GFAP, seen by labeled process. The LPS-treated retinas showed a similar GFAP-staining pattern with thin and thick GFAP-positive Müller cell processes spanning the retina from the inner to the outer part (C, arrows). GCL = ganglion cell layer, INL = inner nuclear layer, IPL = inner plexiform layer, ONL = outer nuclear layer, NFL = nerve fibre layer, OLM = outer limiting membrane. Scale bar: 200 μm.

We utilized GFAP staining to analyze macroglial activation in the retina. Control retinas, at 0 DIV, displayed a very *in vivo*-like expression pattern of GFAP, *i*.*e*. confined to the inner-most retina (Fig 4A). In agreement with numerous reports using the organotypic retinal cultures, we observed increased GFAP expression over time in culture [52]. At 3 DIV, GFAP up-regulation was indeed seen in Müller cell processes spanning the entire retina (Fig 4B). At 4 and 7 DIV its expression was further increased, as judged by labeling of thicker processes compared to 3 DIV (Fig 4D and 4F). At 7 DIV GFAP processes often displayed irregular shapes, compared to earlier time-points (cf. Fig 4F to 4D and 4B). However, quantitative staining intensity measurements did not show a significant difference between time-points in the control retinas. Analysis of GFAP expression after LPS-treatment, showed a similar staining pattern at 3 DIV to that observed in the control group at 4 DIV (Fig 4C), indicating that the LPS treatment induces earlier GFAP expression by Müller cells. Quantitative intensity measurements showed a significant difference between the LPS-treated group and corresponding control only at 3 DIV (4.5±0.7 vs 7.0±0.7 (n = 3/group), p = 0,013). In addition, the GFAP-stained filament pattern showed Müller cell processes continuing throughout the entire retina, *i*.*e*. from inner to outer part, compared to corresponding control retinas. The changed GFAP staining pattern in the LPS groups at 4 and 7 DIV correlated well with the significant changes in gross morphology found within this time period (Fig 1).

Together, changes in filament GFAP staining pattern and expression level was clear in both groups at 3 DIV with the cultivation process itself as well as after additional LPS treatment as stress factor. Our results also show a transient and significant increased macroglial cell response after LPS [60].

### Microglia activation increased after LPS treatment

The hallmarks of activated microglia are altered morphology, migration, proliferation, phagocytosis and release immune mediators [10, 12–14].

#### Microglia activation by cultivation process *per se*

**Fig 5:**
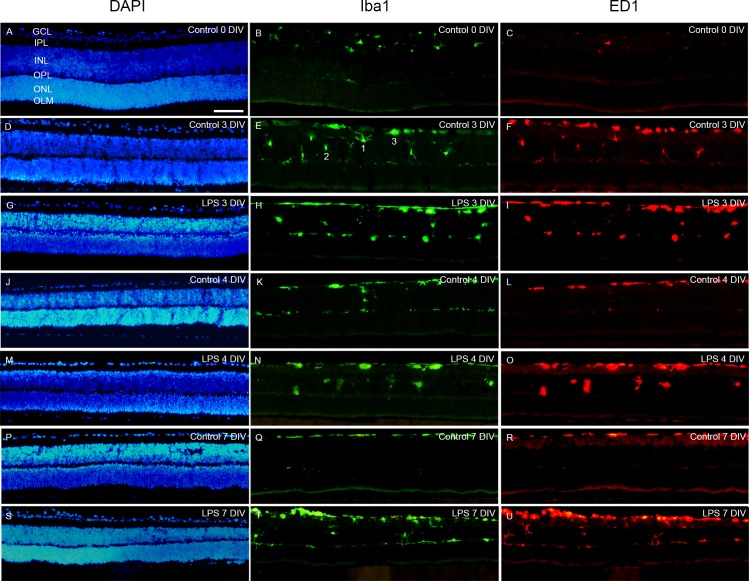
Iba1/ED1- fluorescent immunohistochemistry. Microglial cells were detected using the Iba1 (green) marker, expressed in all microglial stages and ED1 (red) for detecting activated cells. Cells were counterstained with the nuclei marker DAPI (blue). A-C. At 0 DIV, microglial cells were mainly found in the GCL, IPL and OPL, with a small fraction located in the GCL expressing ED1. At 3 and 4 DIV microglial cells were also found in the INL in both LPS-treated retinas and controls (D-F, G-I). At 3, 4 and 7 DIV the majority of the microglia expressed ED1 in both groups. At 7 DIV microglial cells were only found in the INL in the LPS-treated retinas (S-U cf. to P-R). Primarily three morphologies were found and used in the classification of activation stage, *i*.*e*. ramified (see cell #1 in E), round (see cell #2 in E) and amoeboid (see cell #3 in E). GCL = ganglion cell layer, INL = inner nuclear layer, IPL = inner plexiform layer, ONL = outer nuclear layer, NFL = nerve fibre layer, OLM = outer limiting membrane. Scale bar: 200 μm.

First we examined the microglia response induced by the cultivation process. Distribution of microglia was studied using Iba1-labeling. At 0 DIV, similar to the *in vivo* situation at PN7, microglia were located mainly in the two synaptic layers, *i*.*e*. the inner- and outer plexiform layer but also occasionally in the GCL (Fig 5A–5C), as previously reported [52]. A transient presence of microglia was found in the INL at 3 and 4 DIV (Fig 5D–5F).

**Fig 6:**
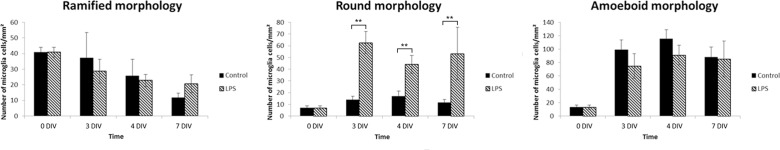
Quantification of microglia morphologies. Iba1-positive microglia was classified into three groups depending on morphology, including ramified, round and amoeboid. ANOVA analysis was performed for comparison between the LPS-treated groups and the control groups. Data are given as mean±StDev (n = 3-6/group), **p<0.01, compared to control at the corresponding time points.

Morphological alterations were then studied and microglia classified based on profiles typical of different activation stages; *i*.*e*. ramified (resting stage), round or amoeboid (activated stages) were quantified [33]. At 0 DIV, the majority displayed a ramified cell profile and about 35% demonstrated round or amoeboid morphologies (Fig 6). At 3, 4 and 7 DIV the majority displayed round or amoeboid morphologies, and the ramified cells decreased over time in culture (Fig 6). At all time-points amoeboid microglia were only found within the GCL. Round microglia was found in the IPL, INL and OPL. At 7 DIV and only very occasionally, microglial processes were found to extend into the ONL.

**Fig 7:**
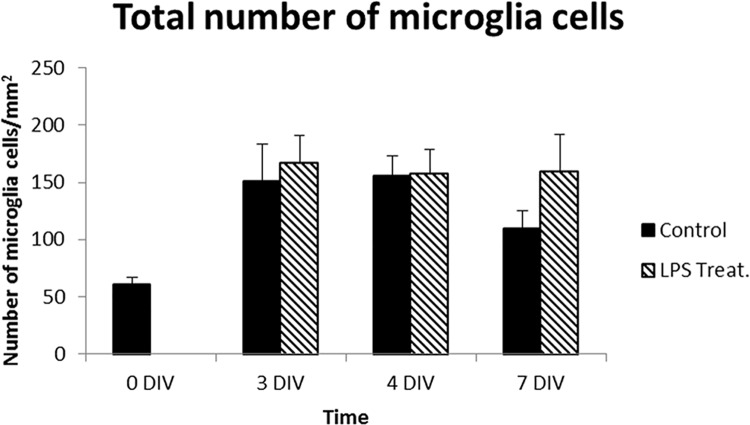
Total numbers of microglia cells. The graph shows quantification of total numbers of Iba1/ED1-positive cells. ANOVA analysis was performed for comparison between the LPS-treated group and the control groups. Data are given as mean ±StDev (n = 3-6/group).

Total number of microglia in controls was examined by quantifying numbers of Iba1-positive cells, and showed significant larger numbers of microglia at all time-points after seeding (Fig 7). In accordance, microglia proliferation has been reported elsewhere to occur within the first days of retinal organotypic cultures [61]. Here, at 7 DIV the number of Iba1-positive cells was smaller than at 3 and 4 DIV, but yet the double compared to at 0 DIV.

**Fig 8:**
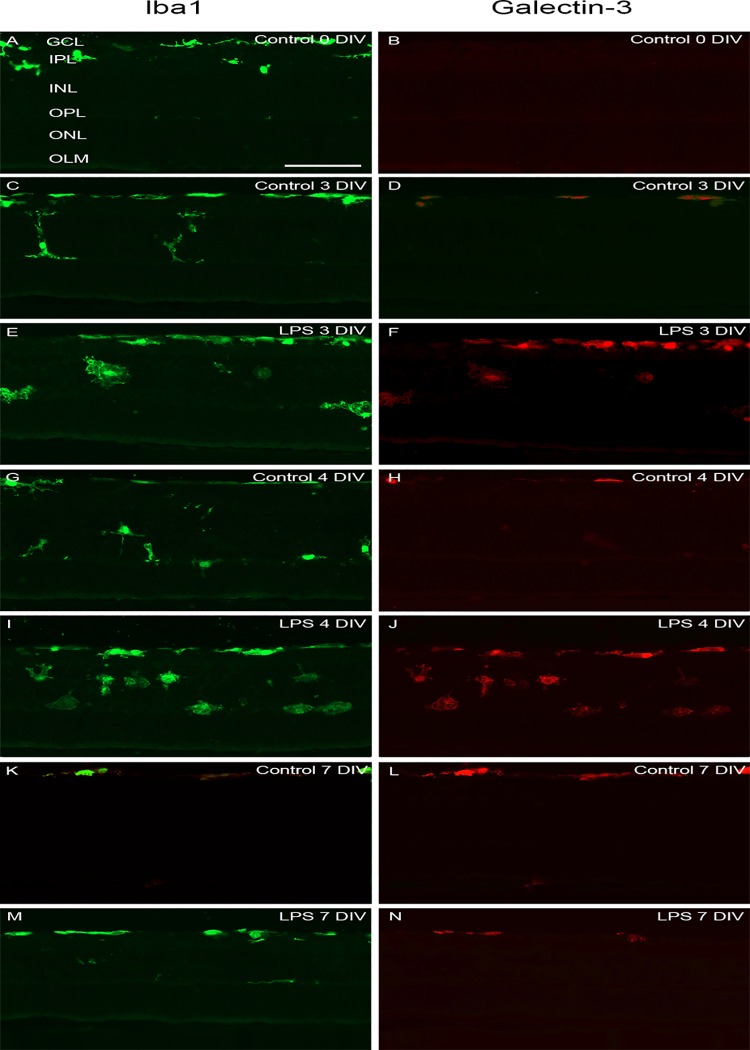
Gal-3 expression in microglia cells. Immunohistochemical staining of Gal-3 (red) expressing microglia. All microglia were detected using the microglia marker Iba1 (green). At 0 DIV no Gal-3-expressing cells were found (A, B). In controls, at 3, 4 and 7 DIV Iba1/Gal-3 co-expressing cells were found and only in the GCL (C, D, G, H, K, and L). LPS-treated retinas displayed larger numbers of Iba1/Gal-3 co-expressing cells that were located in the GCL, INL and OPL at 3, 4 and 7 DIV. Scale bar: 200 μm.

**Fig 9:**
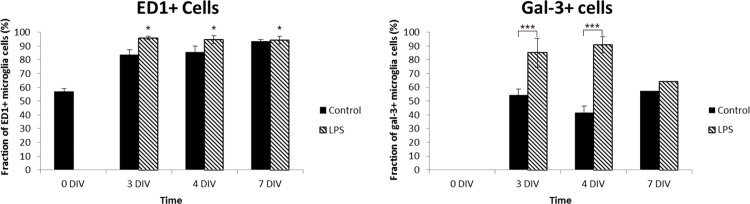
Quantification of ED1 and Gal-3-positive microglial cells. Quantification of fractions of cells expressing ED1 and Gal-3, respectively, of the total number of Iba1-stained cells. ANOVA analysis was performed for comparison between the LPS-treated group and the control groups. Data are expressed as mean±StDev (n = 3/group) *p<0.05, ***p<0.001.

Level of microglia activation was further assessed using double immunohistochemistry with Iba1 and the markers ED1 and Gal-3, respectively [34] (Figs 5 and 8). ED1 is known to be expressed in microglia cells in a phagocytic stage [62]. At 0 DIV, about 50% of the microglial cells expressed ED1 and were primarily found in the GCL and INL (Fig 5). Then, significant larger numbers Iba1/ED1-positive cells as well as increasing with time were found (Fig 9). Gal-3 is a member of a carbohydrate-binding protein family involved in cell activation and inflammation [18], and reported by us to contribute to the full activation of the LPS receptor toll-like receptor 4 (TLR4)) [19]. At 0 DIV no Iba1/Gal-3 labeled cells were found (Figs 8A, 8B and 9). However, at 3, 4 and 7 DIV, equal and large fractions of Iba1-labeled cells co-expressed Gal-3, with the vast majority of these cells located in the GCL (Fig 8C, 8D, 8G, 8H, 8K and 8L). The literature is very sparse on Gal-3 expression in the normal retina, with only one report describing a low level of Gal-3 expression localized to Müller cells at post-natal day 2 in the porcine retina [63]. Here, the vast majority of the Gal-3-expressing cell bodies and processes co-localized with the Iba-1 staining (Fig 8).

Gal-3 levels are increased in several conditions including encephalomyelitis, traumatic brain injury, experimental allergic encephalitis (EAE) and ischemic brain injury [2, 25], and in the retina after ischemia [30]. Gal-3 is found both intra- (in cytoplasm and nucleus) and extracellularly in different cell types and is suggested to play both pro-inflammatory and anti-inflammatory roles which depend on the cell type and insult provided. The pro-inflammatory mechanism was recently described by us, with Gal-3 acting as a ligand by binding to the LPS receptor TLR4, via its CRD domain, and thereby propagating inflammation [19]. In the present study no Gal-3 expression could be detected using immunohistochemistry at 0 DIV, *i*.*e*. PN day7.

**Fig 10:**
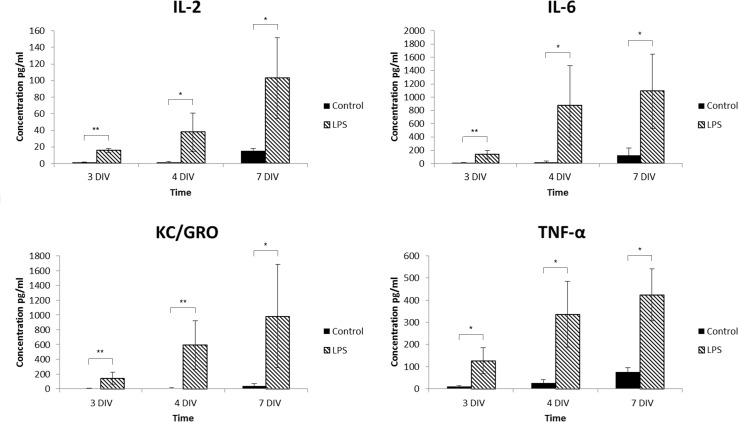
Cytokine release profiles. In collected conditioned media at 3, 4 and 7 DIV, the immune mediator release profile was analyzed using a biochemical assay. Ten well-described cytokines were included; interferon gamma (IFN-γ), interleukin-1β (IL-1β), interleukin-2 (IL-2), interleukin-4 (IL-4), interleukin-5 (IL-5), interleukin-6 (IL-6), interleukin-10 (IL-10), interleukin-12p70 (IL-12p70), KC/GRO and tumour necrosis factor alpha (TNF-α). No detectable levels of release of any of the mediators analyzed were found in controls at any of the time-points included. However, after LPS stimulation at 3 DIV, a significant increase of four of the mediators was found, *i*.*e*. IL-2, IL-6, KC/GRO and TNF-α. Note that the content of these factors further increased over time in culture to higher levels compared to at 3 DIV. Filled bar = control group, Striped bar = LPS group. ANOVA analysis was performed for comparison between the LPS treated group and the control groups. Data are expressed as mean±StDev (n = 3/group), *p<0.05, **p<0.01.

Release of immune mediator production is a well-described criterion for microglia activation; hence, we also biochemically examined the release of a battery of pro-inflammatory mediators. Only at 7 DIV very low concentrations of TNF-α, IL-2, IL-6 and KC/GRO were found in the controls (Fig 10). *In vivo* and in some *in vitro* studies IL-1β is typically released early following injury or during inflammation [64, 65]. Here, we cannot exclude the early release of IL-1β since the earliest time-point studied was 2 DIV. In addition, IL-1β is released from astroglia, reported by IL-1β co-localization with GFAP-staining, but not with CD11b, a microglia/macrophage marker, suggesting that astrocytes are a source of IL-1β release under these conditions [66].

Last we made correlation analysis of microglia behavior and level of apoptosis over time, and found a significant correlation between total numbers of microglia (*i*.*e*. Iba1-positive) and total numbers of TUNEL-positive cells (0,976, p<0.05). In addition, apoptotic cell numbers in the GCL correlated with numbers of amoeboid cells, which were only found in the GCL (0.996, p<0.01).

Altogether, the cultivation *per se* stimulates microglia to an increased activation level compared to at 0 DIV, demonstrated by: transient migration, increase in microglial cell number, morphological changes and increased expression of the markers ED1 and Gal-3. We judge the increase in cell numbers as an evidence of cell proliferation of the resident microglia population, since no macrophages in the here used *in vitro* model could be recruited from the blood circulation.

Notably, a smaller fraction of the microglia expressed Gal-3 than ED1 at 3, 4 and 7 DIV, suggesting different regulation of different fractions of microglia in our model. Furthermore, Gal-3 was not expressed at 0 DIV, which may indicate that this modulator is not involved in the normal microglia regulation, stimulated by the remodeling in the developing retina. Gal-3 is most likely induced in response to the acute massive apoptosis in the GCL caused by the axotomy of the ON. Indeed, it was also in the innermost retina that Iba1/Gal-3 labelled cells were found. The migration of microglia into the INL, from 0 to 3 DIV, may have been stimulated by the normal occurring apoptosis in this layer at 0 DIV.

#### Elevated microglia activation after LPS-treatment

Distribution analysis in LPS-treated retinas revealed Iba1/ED1-positive cells in the GCL, IPL, INL and OPL (Figs 5 and 9). In LPS-treated retinas also cluster formation of microglia was detected, which has been described as a feature of activation in studies using the explanted retina [67]. We believe that the soluble factors released most likely were produced in the microglial cells since these were activated rapidly after LPS stimulation, whereas the macroglial cell activation was not as prominent. In addition, the fraction of round microglia morphologies was larger at all time-points compared to corresponding controls, with an accompanied decrease in fraction of amoeboid cells (Fig 6).

LPS-treatment immediately increased microglial activation. At 3 DIV, a significant increase in the production of TNF-α, IL-2, IL-6 and KC/GRO was found, and a substantial further increase in expression levels over time in culture (Fig 10). In agreement, others have reported release of high levels of the factors TNF-α [32, 33] and IL-6 [33] after LPS stimulation of cultured retina or microglial cell cultures. However, in contrast to our findings, Mertsch *et al*. reported that TNF-α, and IL-6 release activities were reversed to controls levels 3 days after LPS removal in cultured retina [33]. IL-6 increase has been reported to be involved in retina regeneration, and especially RGC regeneration [68]. Here, thus, the absence of increase apoptosis in the GCL may be explained by local and high levels of IL-6 [33]. IL-2 was reported up-regulated in diabetic retinopathy models [59]. KC/GRO, also known as CXCL1, is expressed by Müller cells, microglia and retinal pigment epithelium cells, and has been found expressed in a model of light induced photoreceptor degeneration [69].

Total cell numbers in LPS-treated retinas maintained similar or the same compared to controls all time-points (Fig 7). Similar to corresponding controls, no Ki67 immune-positive cells could be detected (data not shown).

Further analysis of microglia activation level by examination of the markers ED1 and Gal-3, revealed large numbers of phagocytic cell in the INL at 4 DIV, that may correlate with the peak in apoptosis seen in this layer at 3 DIV, in the LPS-treated group (cf Figs 3, 5 and 9). Likewise, more frequent than in corresponding controls, Iba1/ED1-labeled microglia in the OPL were seen extending long processes into the ONL and occasionally the microglial cell had migrated into the ONL, correlating with the increased apoptosis in the ONL at 4 and 7 DIV in the LPS group. Migration of microglia into damaged tissue areas, has been recently reported [70]. Round microglia are known to be the most mobile [71], explaining the distribution of more cells into additional retinal layers compared to controls. In addition, the Gal-3 was always expressed in these round cells, and has indeed been reported to have a potential role in the cell motility [72]. Furthermore, Gal-3 was expressed in a higher number of microglia at both 3 and 4 DIV after LPS administration, but returned to the same level as the control at 7 DIV (Fig 9). We always found Gal-3 labeling co-localized with the Iba1-staining. The vast majority of the Gal-3 expression co-localized with the Iba1-staining and was found in microglia located in the GCL, INL and OPL, and typically in large cells or clusters of cells (Fig 8E, 8F, 8I and 8J).

Correlation analysis in LPS-treated retinas on microglia activation and level of apoptosis revealed a strong correlation in overall level of apoptosis and total numbers of microglia (R^2^ = 0,998, p<0.01). Moreover, the level of apoptosis in the GCL as well as in the INL correlated with decreased fraction of ramified microglia (R^2^ = 0.965, p<0.05), while the level of apoptosis correlated with fraction of round morphologies in the ONL (R^2^ = 0.999, p<0.001). Lastly, we found that the levels of the cytokines IL-2 and IL-6 correlated with the fraction of microglia with an amoeboid profile (R^2^ = 0,989 (p<0.05) and R^2^ = 0.952 (p<0.05), respectively).

## Conclusions

In this study we have demonstrated a cytotoxic *in vivo*-resembling retinal neuroinflammation by challenging organotypic cultured retinas with LPS. In parallel with previous findings we demonstrate that the current cultivation model can elicit activation of an immune competent system in the retina, especially microglia activation. Exposure to LPS was needed to establish a more *in vivo*-like immune response including typical immune mediator release [32, 33].

For the first time, we show a broad cytokine profile release profile within an organotypic retina system with TNF-α, and IL-6 release as previously seen in other studies *in vivo* and *in vitro*. Our model is further characterized by IL-2 and KC/GRO (CXCL1) up-regulation, but no increase of IL-1β, IL-4, IL-5, IL-10, and IL-12. In addition, we show the up-regulation of Gal-3 in a subpopulation of the microglia upon further stimulation. Last we show a strong correlation of the level of microglia activation with level of apoptosis, especially in the LPS challenged retinas.

The concept of neuroinflammation is today a generally accepted idea in retina research, however the mechanisms by which this occurs and consequences of inflammation are still under debate. Lately, it was proposed that changes in cytokine signaling may occur before neuronal cell death, and such findings could open the door for potential therapies that focus on restoring and maintaining function rather than intervening directly with the causes of retinal degeneration. Ultimately, detection of microglial activation level using a biomarker such as Gal-3 may have value in early disease diagnosis, where modulation of microglial responses may alter disease progression.

## References

[1] Hirsch EC, Vyas S, Hunot S. Neuroinflammation in Parkinson's disease. Parkinsonism & related disorders. 2012;18 Suppl 1:S210–2. 22166438 10.1016/S1353-8020(11)70065-7

[2] Pajoohesh-Ganji A, Knoblach SM, Faden AI, Byrnes KR. Characterization of inflammatory gene expression and galectin-3 function after spinal cord injury in mice. Brain Res. 2012;1475:96–105. 10.1016/j.brainres.2012.07.058 22884909 PMC3433585

[3] Zhang F, Jiang L. Neuroinflammation in Alzheimer's disease. Neuropsychiatric disease and treatment. 2015;11:243–56. 10.2147/NDT.S75546 25673992 PMC4321665

[4] Karlstetter M, Scholz R, Rutar M, Wong WT, Provis JM, Langmann T. Retinal microglia: just bystander or target for therapy? Progress in retinal and eye research. 2015;45:30–57. 10.1016/j.preteyeres.2014.11.004 25476242

[5] Liu G, Meng C, Pan M, Chen M, Deng R, Lin L, et al Isolation, purification, and cultivation of primary retinal microvascular pericytes: a novel model using rats. Microcirculation. 2014;21(6):478–89. 10.1111/micc.12121 24495210

[6] Pfister F, Przybyt E, Harmsen MC, Hammes HP. Pericytes in the eye. Pflugers Arch. 2013;465(6):789–96. 10.1007/s00424-013-1272-6 23568370

[7] Caspi RR. A look at autoimmunity and inflammation in the eye. The Journal of clinical investigation. 2010;120(9):3073–83. 10.1172/JCI42440 20811163 PMC2929721

[8] Wang AL, Yu AC, Lau LT, Lee C, Wu le M, Zhu X, et al Minocycline inhibits LPS-induced retinal microglia activation. Neurochem Int. 2005;47(1–2):152–8. 15904993 10.1016/j.neuint.2005.04.018

[9] Whitcup SM, Nussenblatt RB, Lightman SL, Hollander DA. Inflammation in retinal disease. International journal of inflammation. 2013;2013:724648 10.1155/2013/724648 24109539 PMC3784265

[10] Bosco A, Steele MR, Vetter ML. Early microglia activation in a mouse model of chronic glaucoma. J Comp Neurol. 2011;519(4):599–620. 10.1002/cne.22516 21246546 PMC4169989

[11] Gupta N, Yucel YH. Glaucoma as a neurodegenerative disease. Curr Opin Ophthalmol. 2007;18(2):110–4. 17301611 10.1097/ICU.0b013e3280895aea

[12] Johnson EC, Morrison JC. Friend or foe? Resolving the impact of glial responses in glaucoma. Journal of glaucoma. 2009;18(5):341–53. 10.1097/IJG.0b013e31818c6ef6 19525723 PMC2697444

[13] Langmann T. Microglia activation in retinal degeneration. Journal of leukocyte biology. 2007;81(6):1345–51. 17405851 10.1189/jlb.0207114

[14] Madeira MH, Boia R, Santos PF, Ambr, #xf3, sio A, et al Contribution of Microglia-Mediated Neuroinflammation to Retinal Degenerative Diseases. Mediators of Inflammation. 10.1155/2015/673090 PMC4385698 25873768

[15] Nickells RW. From ocular hypertension to ganglion cell death: a theoretical sequence of events leading to glaucoma. Canadian journal of ophthalmology Journal canadien d'ophtalmologie. 2007;42(2):278–87. 17392853

[16] Soto I, Howell GR. The complex role of neuroinflammation in glaucoma. Cold Spring Harbor perspectives in medicine. 2014;4(8). 10.1101/cshperspect.a017269 PMC4109578 24993677

[17] Wilson GN, Inman DM, Dengler Crish CM, Smith MA, Crish SD. Early pro-inflammatory cytokine elevations in the DBA/2J mouse model of glaucoma. J Neuroinflammation. 2015;12:176 10.1186/s12974-015-0399-0 26376776 PMC4574349

[18] Boza-Serrano A, Reyes JF, Rey NL, Leffler H, Bousset L, Nilsson U, et al The role of Galectin-3 in alpha-synuclein-induced microglial activation. Acta Neuropathol Commun. 2014;2:156 10.1186/s40478-014-0156-0 25387690 PMC4236422

[19] Burguillos MA, Svensson M, Schulte T, Boza-Serrano A, Garcia-Quintanilla A, Kavanagh E, et al Microglia-Secreted Galectin-3 Acts as a Toll-like Receptor 4 Ligand and Contributes to Microglial Activation. Cell reports. 2015. 10.1016/j.celrep.2015.02.012 25753426

[20] Canning P, Glenn JV, Hsu DK, Liu FT, Gardiner TA, Stitt AW. Inhibition of advanced glycation and absence of galectin-3 prevent blood-retinal barrier dysfunction during short-term diabetes. Exp Diabetes Res. 2007;2007:51837 17641742 10.1155/2007/51837 PMC1880865

[21] Uehara F, Ohba N, Ozawa M. Isolation and characterization of galectins in the mammalian retina. Investigative ophthalmology & visual science. 2001;42(10):2164–72. 11527926

[22] Leffler H, Carlsson S, Hedlund M, Qian Y, Poirier F. Introduction to galectins. Glycoconj J. 2004;19(7–9):433–40. 10.1023/B:GLYC.0000014072.34840.04 14758066

[23] Seetharaman J, Kanigsberg A, Slaaby R, Leffler H, Barondes SH, Rini JM. X-ray crystal structure of the human galectin-3 carbohydrate recognition domain at 2.1-A resolution. J Biol Chem. 1998;273(21):13047–52. 9582341 10.1074/jbc.273.21.13047

[24] Jeon SB, Yoon HJ, Chang CY, Koh HS, Jeon SH, Park EJ. Galectin-3 exerts cytokine-like regulatory actions through the JAK-STAT pathway. J Immunol. 2010;185(11):7037–46. 10.4049/jimmunol.1000154 20980634

[25] Jiang HR, Al Rasebi Z, Mensah-Brown E, Shahin A, Xu D, Goodyear CS, et al Galectin-3 deficiency reduces the severity of experimental autoimmune encephalomyelitis. J Immunol. 2009;182(2):1167–73. 19124760 10.4049/jimmunol.182.2.1167

[26] Karlsson A, Christenson K, Matlak M, Bjorstad A, Brown KL, Telemo E, et al Galectin-3 functions as an opsonin and enhances the macrophage clearance of apoptotic neutrophils. Glycobiology. 2009;19(1):16–20. 10.1093/glycob/cwn104 18849325

[27] Lepur A, Carlsson MC, Novak R, Dumic J, Nilsson UJ, Leffler H. Galectin-3 endocytosis by carbohydrate independent and dependent pathways in different macrophage like cell types. Biochim Biophys Acta. 2012;1820(7):804–18. 10.1016/j.bbagen.2012.02.018 22450157

[28] Sano H, Hsu DK, Apgar JR, Yu L, Sharma BB, Kuwabara I, et al Critical role of galectin-3 in phagocytosis by macrophages. The Journal of clinical investigation. 2003;112(3):389–97. 12897206 10.1172/JCI17592 PMC166291

[29] Shin T. The pleiotropic effects of galectin-3 in neuroinflammation: a review. Acta Histochem. 2013;115(5):407–11. 10.1016/j.acthis.2012.11.010 23305876

[30] Manouchehrian O, Arner K, Deierborg T, Taylor L. Who let the dogs out?: detrimental role of Galectin-3 in hypoperfusion-induced retinal degeneration. J Neuroinflammation. 2015;12:92 10.1186/s12974-015-0312-x 25968897 PMC4490716

[31] Nguyen JV, Soto I, Kim KY, Bushong EA, Oglesby E, Valiente-Soriano FJ, et al Myelination transition zone astrocytes are constitutively phagocytic and have synuclein dependent reactivity in glaucoma. Proc Natl Acad Sci U S A. 2011;108(3):1176–81. 10.1073/pnas.1013965108 21199938 PMC3024691

[32] Ferrer-Martin RM, Martin-Oliva D, Sierra A, Carrasco MC, Martin-Estebane M, Calvente R, et al Microglial cells in organotypic cultures of developing and adult mouse retina and their relationship with cell death. Exp Eye Res. 2014;121:42–57. 10.1016/j.exer.2014.02.015 24582572

[33] Mertsch K, Hanisch UK, Kettenmann H, Schnitzer J. Characterization of microglial cells and their response to stimulation in an organotypic retinal culture system. J Comp Neurol. 2001;431(2):217–27. 11170001

[34] Ito D, Tanaka K, Suzuki S, Dembo T, Fukuuchi Y. Enhanced expression of Iba1, ionized calcium-binding adapter molecule 1, after transient focal cerebral ischemia in rat brain. Stroke; a journal of cerebral circulation. 2001;32(5):1208–15. 11340235 10.1161/01.str.32.5.1208

[35] Lossi L, Alasia S, Salio C, Merighi A. Cell death and proliferation in acute slices and organotypic cultures of mammalian CNS. Prog Neurobiol. 2009;88(4):221–45. 10.1016/j.pneurobio.2009.01.002 19552996

[36] Paquet-Durand F, Hauck SM, van Veen T, Ueffing M, Ekstrom P. PKG activity causes photoreceptor cell death in two retinitis pigmentosa models. J Neurochem. 2009;108(3):796–810. 10.1111/j.1471-4159.2008.05822.x 19187097

[37] Vinet J, Weering HR, Heinrich A, Kalin RE, Wegner A, Brouwer N, et al Neuroprotective function for ramified microglia in hippocampal excitotoxicity. J Neuroinflammation. 2012;9:27 10.1186/1742-2094-9-27 22293457 PMC3292937

[38] Dunlop SA, Beazley LD. Cell death in the developing retinal ganglion cell layer of the wallaby Setonix brachyurus. J Comp Neurol. 1987;264(1):14–23. 3680622 10.1002/cne.902640103

[39] McKernan DP, Caplis C, Donovan M, O'Brien C J, Cotter TG. Age-dependent susceptibility of the retinal ganglion cell layer to cell death. Investigative ophthalmology & visual science. 2006;47(3):807–14. 16505011 10.1167/iovs.05-0520

[40] Engelsberg K, Ehinger B, Wasselius J, Johansson K. Apoptotic cell death and microglial cell responses in cultured rat retina. Graefes Arch Clin Exp Ophthalmol. 2004;242(3):229–39. 14745560 10.1007/s00417-003-0780-z

[41] Ferrer-Martin RM, Martin-Oliva D, Sierra-Martin A, Carrasco MC, Martin-Estebane M, Calvente R, et al Microglial Activation Promotes Cell Survival in Organotypic Cultures of Postnatal Mouse Retinal Explants. PLoS One. 2015;10(8):e0135238 10.1371/journal.pone.0135238 26252475 PMC4529135

[42] Chen ZH, Jalabi W, Shpargel KB, Farabaugh KT, Dutta R, Yin XH, et al Lipopolysaccharide-Induced Microglial Activation and Neuroprotection against Experimental Brain Injury Is Independent of Hematogenous TLR4. Journal of Neuroscience. 2012;32(34):11706–15. 10.1523/JNEUROSCI.0730-12.2012 22915113 PMC4461442

[43] Skelly DT, Hennessy E, Dansereau MA, Cunningham C. A systematic analysis of the peripheral and CNS effects of systemic LPS, IL-1beta, [corrected] TNF-alpha and IL-6 challenges in C57BL/6 mice. PLoS One. 2013;8(7):e69123 10.1371/journal.pone.0069123 23840908 PMC3698075

[44] Sriram K, Miller DB, O'Callaghan JP. Minocycline attenuates microglial activation but fails to mitigate striatal dopaminergic neurotoxicity: role of tumor necrosis factor-alpha. J Neurochem. 2006;96(3):706–18. 16405514 10.1111/j.1471-4159.2005.03566.x

[45] Block ML, Zecca L, Hong JS. Microglia-mediated neurotoxicity: uncovering the molecular mechanisms. Nat Rev Neurosci. 2007;8(1):57–69. 17180163 10.1038/nrn2038

[46] Farinelli P, Perera A, Arango-Gonzalez B, Trifunovic D, Wagner M, Carell T, et al DNA methylation and differential gene regulation in photoreceptor cell death. Cell death & disease. 2014;5:e1558. 25476906 10.1038/cddis.2014.512 PMC4649831

[47] Paquet-Durand F, Silva J, Talukdar T, Johnson LE, Azadi S, van Veen T, et al Excessive activation of poly(ADP-ribose) polymerase contributes to inherited photoreceptor degeneration in the retinal degeneration 1 mouse. J Neurosci. 2007;27(38):10311–9. 17881537 10.1523/JNEUROSCI.1514-07.2007 PMC6672664

[48] Sanyal S, Bal AK. Comparative light and electron microscopic study of retinal histogenesis in normal and rd mutant mice. Z Anat Entwicklungsgesch. 1973;142(2):219–38. 4781863 10.1007/BF00519723

[49] Richard S. Smith SWMJ, Patsy M. Nishina, John P. Sundberg. Systematic Evaluation of the Mouse Eye: Anatomy, Pathology, and Biomethod. Systematic Evaluation of the Mouse Eye: Anatomy, Pathology, and Biomethods. Research Methods For Mutant Mice CRC Press; 2001. p. 195–226.

[50] Caffe AR, Ahuja P, Holmqvist B, Azadi S, Forsell J, Holmqvist I, et al Mouse retina explants after long-term culture in serum free medium. J Chem Neuroanat. 2001;22(4):263–73. 11719023 10.1016/s0891-0618(01)00140-5

[51] Ankarcrona M, Dypbukt JM, Bonfoco E, Zhivotovsky B, Orrenius S, Lipton SA, et al Glutamate-induced neuronal death: a succession of necrosis or apoptosis depending on mitochondrial function. Neuron. 1995;15(4):961–73. 7576644 10.1016/0896-6273(95)90186-8

[52] Soderstjerna E, Bauer P, Cedervall T, Abdshill H, Johansson F, Johansson UE. Silver and gold nanoparticles exposure to in vitro cultured retina—studies on nanoparticle internalization, apoptosis, oxidative stress, glial- and microglial activity. PLoS One. 2014;9(8):e105359 10.1371/journal.pone.0105359 25144684 PMC4140780

[53] Ahl M, Avdic U, Skoug C, Ali I, Chugh D, Johansson UE, et al Immune response in the eye following epileptic seizures. J Neuroinflammation. 2016;13(1):155 10.1186/s12974-016-0618-3 27346214 PMC4922060

[54] Amor S, Puentes F, Baker D, van der Valk P. Inflammation in neurodegenerative diseases. Immunology. 2010;129(2):154–69. 10.1111/j.1365-2567.2009.03225.x 20561356 PMC2814458

[55] Fakhoury M. Role of Immunity and Inflammation in the Pathophysiology of Neurodegenerative Diseases. Neuro-degenerative diseases. 2015;15(2):63–9. 10.1159/000369933 25591815

[56] Berkelaar M, Clarke DB, Wang YC, Bray GM, Aguayo AJ. Axotomy results in delayed death and apoptosis of retinal ganglion cells in adult rats. J Neurosci. 1994;14(7):4368–74. 8027784 10.1523/JNEUROSCI.14-07-04368.1994 PMC6577016

[57] Hakansson G, Gesslein B, Gustafsson L, Englund-Johansson U, Malmsjo M. Hypoxia-inducible factor and vascular endothelial growth factor in the neuroretina and retinal blood vessels after retinal ischemia. Journal of ocular biology, diseases, and informatics. 2010;3(1):20–9. 10.1007/s12177-010-9050-6 21139705 PMC2956450

[58] Guerin CJ, Anderson DH, Fisher SK. Changes in intermediate filament immunolabeling occur in response to retinal detachment and reattachment in primates. Investigative ophthalmology & visual science. 1990;31(8):1474–82. 2387680

[59] Reichenbach A, Bringmann A. New functions of Muller cells. Glia. 2013;61(5):651–78. 10.1002/glia.22477 23440929

[60] Jang S, Lee JH, Choi KR, Kim D, Yoo HS, Oh S. Cytochemical alterations in the rat retina by LPS administration. Neurochem Res. 2007;32(1):1–10. 17160463 10.1007/s11064-006-9215-7

[61] Schnitzer J, Scherer J. Microglial cell responses in the rabbit retina following transection of the optic nerve. J Comp Neurol. 1990;302(4):779–91. 1964466 10.1002/cne.903020410

[62] Bauer J, Sminia T, Wouterlood FG, Dijkstra CD. Phagocytic activity of macrophages and microglial cells during the course of acute and chronic relapsing experimental autoimmune encephalomyelitis. J Neurosci Res. 1994;38(4):365–75. 7932870 10.1002/jnr.490380402

[63] Kim J, Moon C, Ahn M, Joo HG, Jin JK, Shin T. Immunohistochemical localization of galectin-3 in the pig retina during postnatal development. Mol Vis. 2009;15:1971–6. 19816601 PMC2756516

[64] Hopkins SJ, Rothwell NJ. Cytokines and the nervous system. I: Expression and recognition. Trends Neurosci. 1995;18(2):83–8. 7537419

[65] Merrill JE, Benveniste EN. Cytokines in inflammatory brain lesions: helpful and harmful. Trends Neurosci. 1996;19(8):331–8. 8843602 10.1016/0166-2236(96)10047-3

[66] Ren K, Torres R. Role of interleukin-1beta during pain and inflammation. Brain research reviews. 2009;60(1):57–64. 10.1016/j.brainresrev.2008.12.020 19166877 PMC3076185

[67] Streit WJ, Walter SA, Pennell NA. Reactive microgliosis. Prog Neurobiol. 1999;57(6):563–81. 10221782 10.1016/s0301-0082(98)00069-0

[68] Leibinger M, Muller A, Gobrecht P, Diekmann H, Andreadaki A, Fischer D. Interleukin-6 contributes to CNS axon regeneration upon inflammatory stimulation. Cell death & disease. 2013;4:e609. 23618907 10.1038/cddis.2013.126 PMC3641349

[69] Rutar M, Natoli R, Chia R, Valter K, Provis JM. Chemokine-mediated inflammation in the degenerating retina is coordinated by Muller cells, activated microglia, and retinal pigment epithelium. J Neuroinflammation. 2015;12(1):8. 25595590 10.1186/s12974-014-0224-1 PMC4308937

[70] Brockhaus J, Moller T, Kettenmann H. Phagocytozing ameboid microglial cells studied in a mouse corpus callosum slice preparation. Glia. 1996;16(1):81–90. 8787776 10.1002/(SICI)1098-1136(199601)16:1<81::AID-GLIA9>3.0.CO;2-E

[71] Kettenmann H, Hanisch UK, Noda M, Verkhratsky A. Physiology of microglia. Physiol Rev. 2011;91(2):461–553. 10.1152/physrev.00011.2010 21527731

[72] Comte I, Kim Y, Young CC, van der Harg JM, Hockberger P, Bolam PJ, et al Galectin-3 maintains cell motility from the subventricular zone to the olfactory bulb. J Cell Sci. 2011;124(Pt 14):2438–47. 10.1242/jcs.079954 21693585 PMC3124373

